# Tumor Dormancy and Interplay with Hypoxic Tumor Microenvironment

**DOI:** 10.3390/ijms20174305

**Published:** 2019-09-03

**Authors:** Elena Butturini, Alessandra Carcereri de Prati, Diana Boriero, Sofia Mariotto

**Affiliations:** Department of Neuroscience, Biomedicine and Movement Sciences, Section of Biological Chemistry, University of Verona, 37134 Verona, Italy (E.B.) (A.C.d.P.) (D.B.)

**Keywords:** dormancy, tumor microenvironment, hypoxia

## Abstract

The tumor microenvironment is a key factor in disease progression, local resistance, immune-escaping, and metastasis. The rapid proliferation of tumor cells and the aberrant structure of the blood vessels within tumors result in a marked heterogeneity in the perfusion of the tumor tissue with regions of hypoxia. Although most of the tumor cells die in these hypoxic conditions, a part of them can adapt and survive for many days or months in a dormant state. Dormant tumor cells are characterized by cell cycle arrest in G0/G1 phase as well as a low metabolism, and are refractive to common chemotherapy, giving rise to metastasis. Despite these features, the cells retain their ability to proliferate when conditions improve. An understanding of the regulatory machinery of tumor dormancy is essential for identifying early cancer biomarkers and could provide a rationale for the development of novel agents to target dormant tumor cell populations. In this review, we examine the current knowledge of the mechanisms allowing tumor dormancy and discuss the crucial role of the hypoxic microenvironment in this process.

## 1. Introduction

The tumor mass is composed not only by cancer cells but also by a variety of resident and infiltrating host cells, secreted factors and extracellular matrix components, known as the tumor microenvironment (TME). Tumor progression is influenced by interactions of cancer cells with their environment that determine if the primary tumor is eradicated, metastasizes, or establishes dormant micro-metastases. The TME can also influence therapeutic responses, justifying the recent research studies on new therapeutic approaches that target components of the TME.

## 2. Tumor Microenvironment

**Figure 1:**
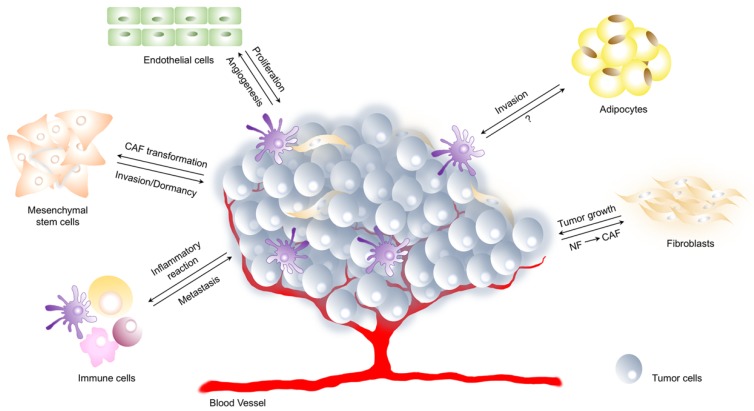
Components of tumor microenvironment. Tumor microenvironment (TME) is a complex network composed of extracellular matrix, cells of hematopoietic origin (immune and inflammatory cells) and cells of mesenchymal origin (fibroblasts, endothelial cells, adipocytes). The interaction between tumor and its microenvironment is crucial for cancer development. Tumor cells promote angiogenesis, induce inflammatory response, stimulate transformation of normal fibroblasts (NF) and mesenchymal stem cells towards cancer-associated fibroblasts (CAF). On the other hand, mesenchymal stem cells as well as immune cells promote and regulate tumor metastasis or dormancy, endothelial cells induce tumor cell proliferation and adipocytes play a crucial role in tumor cell invasion.

TME consists of a complex network of components that regulates tumor initiation, malignant progression and metastasis and has profound effects on therapeutic efficacy [1]. These elements can be widely classified into three main groups: (i) cells of hematopoietic origin, (ii) cells of mesenchymal origin, and (iii) non-cellular components (Figure 1) [2]. First, cells of hematopoietic origin arise in the bone marrow and can be subdivided into cells of lymphoid lineage, consisting of T cells, B cells, natural killer cells, and those of myeloid lineage, which include macrophages, neutrophils and myeloid-derived suppressor cells. The different subsets of cells have either positive or negative effects on the outcome of the tumor [3].

Then, cells of mesenchymal origin derive from the mesenchyme and include fibroblasts, myofibroblasts, mesenchymal stem cells, adipocytes and endothelial cells. Fibroblast, myofibroblast, and mesenchymal stem cells directly support cancer stem cells (CSCs) by creating a favourable niche and facilitating tumor progression, while endothelial cells constitute the walls of blood vessels and have a major role in vascular function and angiogenesis. So far, adipocytes were thought only as energy storage houses while recent studies have revealed the importance of factors secreted by adipocytes in tumor progression [1].

Finally, the extracellular matrix, the major non-cellular component of the TME, is involved in the formation of a stem cell niche. Although it acts to maintain tissue architecture and prevent cancer cells formation, an abnormal extracellular matrix has been shown to promote tumor growth and angiogenesis [4].

Many hallmarks of cancer are related to TME, including the ability to induce proliferation and inhibit apoptosis, to reprogram metabolism, to increase factors that support cancer invasion and metastasis, and to promote angiogenesis. Because the rapid proliferation of the cells requires an accelerated production of basic cellular building blocks, differences in cellular metabolic programs occur within the cells of TME and some proteins, such as HIF-1α, PI3K, AKT, p53, PTEN, known as crucial components in the metabolic pathways, can be differently regulated in cancer cells [5]. Although recent studies have revealed the importance of fatty acids and proteins as fuel sources for cancer cells, aerobic glycolysis remains the major fuel source for tumor cells [6]. It is well described that cancer cells can metabolize 10-fold more glucose than normal tissues to produce lactate. Despite enhanced glycolysis, most cancer cells also maintain mitochondrial respiration to produce a significant amount of ATP. The metabolic switch of cancer cells toward a more glycolytic phenotype increases the concentration of protons [H^+^], causing the acidification of cytoplasm. To overcome low intracellular pH (pHi), cancer cells employ a large redundancy of mechanisms, such as the activation of plasma membrane proton pump ATPase (V-ATPase), carbonic anhydrases (CAIXs) and Na^+^/H^+^ exchangers or anion exchangers that determine a slow increase of pHi. In response to H^+^ efflux, the pH of tumor extracellular space (pHe) becomes acidic, forming a reversed pH gradient. The acidosis of TME promotes cancer progression and induces migration and invasion [7].

Moreover, TME is characterized by disorganized blood vessels that are immature, tortuous and hyperpermeable. The tumor vasculature is typically a complex labyrinth of vessels in which arterioles, capillaries and venules are not clearly identifiable. Furthermore, tumor vessels are more permeable than normal ones because they are poorly invested with smooth muscle cells and have a discontinuous endothelial cell lining with an abnormal basement membrane. Increased vessel permeability results in aberrant osmotic forces, leading to the accumulation of vascular contents and elevated interstitial fluid pressure. As result, the irregular vasculature leads to impaired tumoral blood flow that cannot supply nutrient to cells and remove waste products. In addition, the aberrant geometry of vessels causes an inadequate oxygen supply to tumor cells with hypoxia micro-regional. These particular characteristics of tumor vasculature lead to adverse micro-environmental conditions that obstruct traditional therapeutic anti-cancer strategies [8,9]. Overall, the identification of the most important molecular players in TME represents the first step toward the formulation of new therapeutic cancer treatment.

### 2.1. Hypoxia in Tumor Microenvironment

Due to rapid cell growth and abnormalities of the vasculature network, most solid tumors present disrupted O_2_ homeostasis that results in regions of hypoxia exhibiting oxygenation levels even lower than their tissue of origin [10]. In this microenvironment cells undergo genetic and adaptive changes that allow them to survive and even proliferate.

Based on pathogenesis and time frames three subtypes of tumor hypoxia have been described [11]. Acute hypoxia or ‘perfusion-limited’ hypoxia mainly results from transient perturbation in perfusion (e.g., vascular occlusion by cell aggregates), fluctuating red blood cell fluxes or short-term contractions of the interstitial matrix. The time frame of acute hypoxia is variable in tumors, and it can last from several minutes up to a few days. Acute hypoxia allows cells to survive by activating different mechanisms such as autophagy that is achieved by decreasing oxidative metabolism.

Chronic hypoxia or “diffusion-related” hypoxia is mainly a consequence of a limited diffusion of oxygen with tumor mass expansion. Longer exposure to hypoxia determines long-term cellular changes. Since chronic hypoxia hampers DNA repair systems leading to genetic instability and mutagenesis, it is associated with a high frequency of DNA breaks and the accumulation of DNA replication errors. Moreover, chronic hypoxia induces metabolic switch from oxidative phosphorylation to anaerobic glycolysis that leads to continuous lactate production from pyruvate resulting in acidosis [12]. Although both acute and chronic hypoxia increase resistance to therapy, the latter is associated with a more aggressive tumor phenotype through the development of micro-niches of quiescent cells and induction of spontaneous metastasis [13,14,15].

A third type of hypoxia, called cycling hypoxia, is characterized by oxygen fluctuations with periods of deep hypoxia and moderate hypoxia, and is more commonly associated to hypoxia-reoxygenation profile [16,17]. Cycling hypoxia probably represents the oxygenation state that better reflects in vivo situation. The biological responses activated by reoxygenation can instigate continuous adaptive mechanisms that contribute to increase the aggressiveness of the cancer cells. Some authors report that high level of reactive oxygen species (ROS) induced by cycling hypoxia may contribute to tumor cells survival and progression [16,18]. Thus, the chemoresistance, the radio-resistance and the ability to proliferate or metastasize are extensively enhanced during hypoxia/reoxygenation cycles.

### 2.2. Hypoxia Inducible Factors in Tumor Microenvironment

Hypoxic signaling is mainly mediated via the hypoxia-inducible transcription factors (HIFs), that induce genes involved in angiogenesis, growth and cell survival, energy metabolism, invasion and metastasis.

HIFs are heterodimeric transcription factors consisting in a stable and constitutively expressed β-subunit (HIF-β) and an oxygen sensitive α-subunit (HIF-α), that under normoxia is targeted to proteasomal degradation. Both subunits contain basic helix-loop-helix and PER-ARNT-SIM (PAS) domains that are important for DNA binding and dimerization. The HIF-α subunit also contains an oxygen-dependent degradation domain (ODD) that regulates the oxygen-dependent degradation [19,20,21]. To date, three isoforms of HIF-α subunit (HIF-1α, HIF-2α and HIF-3α) have been described, among which HIF-1α and HIF-2α are the best characterized. While HIF-1α is expressed ubiquitously, HIF-2α is expressed in a more restricted number of cell types such as hepatocytes and endothelial cells. HIF-1α and HIF-2α have a similar C-terminal transactivation domain (C-TAD), which contributes to the transcription of their shared targets. The differences in their N-terminal transactivation domain (N-TAD) confer the different binding capabilities and specificity among their targets [22]. HIF-3α is the most different from the three isoforms and lacks the C-TAD [23].

In the presence of oxygen, conserved proline residues on the α-subunit are hydroxylated promoting its degradation. This hydroxylation is mediated by prolyl-4-hydroxylases (PHDs), oxygen, iron, and ascorbate dependent enzymes belonging to the 2-oxoglutarate-dependent oxygenase superfamily [24]. The hydroxylation of HIF-α allows the rapid interaction with the von Hippel-Lindau tumor-suppression protein (VHL), a component of E3 ubiquitin ligase complex which tags HIF-α for proteasomal degradation.

HIF transcriptional activity is also regulated by a second oxygen-sensitive hydroxylation event mediated by factor inhibiting HIF-1 (FIH-1) [25,26]. FIH-1 is a 2-oxoglutarate-dependent oxygenase that hydroxylate an asparagine residue in the C-TAD of HIF-α, thereby impairing interaction with the p300/CBP co-activator [27].

**Figure 2:**
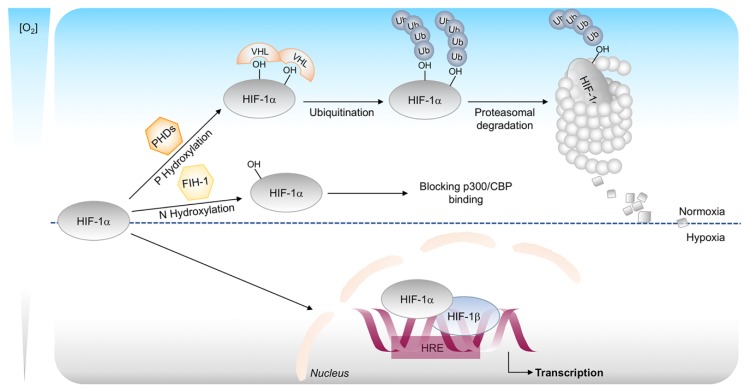
Canonical regulation of HIF-1α stability. In oxygenated conditions, HIF-1α is hydroxylated on proline residues by prolyl-4-hydroxylases (PHDs) and polyubiquitinated by the von Hippel–Lindau protein (pVHL). This leads to degradation of HIF-1α by the 26S proteasome system. The oxygen-dependent hydroxylation of asparagine residue by the enzyme FIH-1 impairs p300 and CBP binding and inhibits HIF-1 mediated gene transcription. In hypoxic conditions, HIF-1α is stabilized and translocated into the nucleus, where it binds to its dimerization partner HIF-1β and enhances the transcription of HIF target genes.

Thus, controlling both the degradation and inactivation of HIF-1α subunit, PHDs and FIHs ensure full repression of the HIF pathway under normoxia. On the other hand, when the oxygen level drops, the hydroxylases lose their activity and HIF-1α is stabilized and translocates into the nucleus where it dimerizes with HIF-1β subunit. HIF complex binds to DNA in the hypoxia-response elements (HRE) region and promotes genes transcription (Figure 2) [28].

In most in vitro chronic hypoxia studies, HIF-1α subunit levels are increased and stabilized within few hours, but after few days decrease to lower expression levels. This is likely due to the HIF-1-mediated up-regulation of PHD2, which retains enough activity to hydroxylate HIF-1α, resulting in its renewed degradation [29]. Cycling hypoxia results in an enhanced activity and stabilization of HIF-1α, which is at a much greater level than that in chronic hypoxia [30]. This damaging phenotype, where HIF-1α is accumulated, is also associated with an increased resistance to radiotherapy and chemotherapy as well as increased metastatic potential [12].

During hypoxia, HIFs support and even enhance the metabolic reprogramming of glycolysis through the up-regulation of almost all glycolytic genes, like hexokinase 1 and 3, aldolase A and C and glyceraldehyde 3-phosphate dehydrogenase-6, and the monocarboxylate transporters that export lactate.

**Figure 3:**
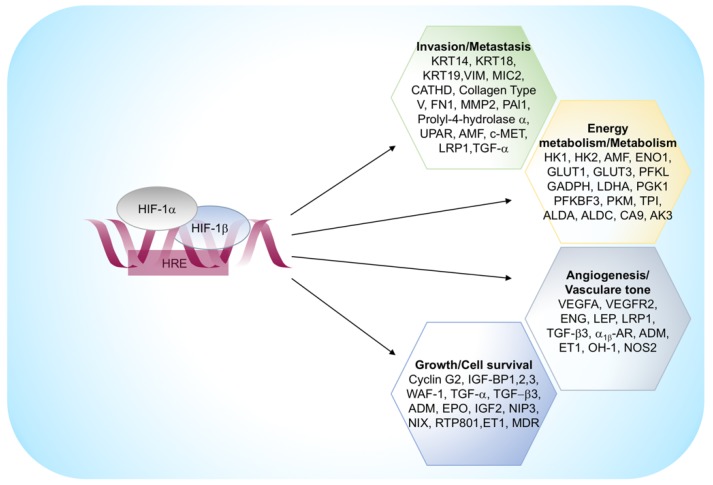
HIF-1α regulated genes. α_1β_ -AR, α_1β_ -adrenergic receptor; ADM, adrenomedullin; ALDA, aldolase A; AK3, adenylate kinase 3; ALDC, aldolase C; AMF, autocrine motility factor; CA 9, carbonic anhydrase 9; CATHD, cathepsin D; ENG, endoglin; ET1, endothelin-1; ENO1, enolase 1; EPO, erythropoietin; FN1, fibronectin 1; GLUT1, glucose transporter 1; GLUT3, glucose transporter 3; GAPDH, glyceraldehyde-3-P-dehydrogenase; HK1, hexokinase 1; HK2, hexokinase 2; IGF2, insulin-like growth-factor 2; IGF-BP1, IGF-factor-binding-protein 1; IGF-BP2, IGF-factor-binding-protein 2; IGF-BP3, IGF-factor-binding-protein 3; KRT14, keratin 14; KRT18, keratin 18; KRT19, keratin 19; LDHA, lactate dehydrogenase A; LEP, leptin; LRP1, Low density lipoprotein receptor-related protein 1; LDL-receptor-related protein 1; MDR1, multidrug resistance 1; MMP2, matrix metalloproteinase 2; NOS2, nitric oxide synthase 2; OH-1, heme oxygenase 1; PAI1, plasminogen-activator inhibitor 1; PFKBF3, 6-phosphofructo-2-kinase/fructose-2,6-biphosphatase-3; PFKL, phosphofructokinase L; PGK 1, phosphoglycerate kinase 1; PKM, pyruvate kinase M; TGF-α, transforming growth factor-α; TGF-β3, transforming growth factor-β3; TPI, triosephosphate isomerase; VEGFA, vascular endothelial growth factor A; UPAR, urokinase plasminogen activator receptor; VEGFR2, VEGF receptor-2; VIM, vimentin.

HIFs also regulate a set of genes involved in extracellular matrix remodeling, migration and digestion of the basement membrane. This group includes vimentine, fibronectine, keratins, matrix metalloproteinase 2, and cathepsin D that is associated with a concomitant loss of E-cadherin, a crucial feature of epithelium-mesenchyme transition [31]. The principal HIF-1α regulated genes are reported in Figure 3.

### 2.3. ROS Production in Tumor Microenvironment

ROS are a broad class of oxygen radical species that are produced in cells as normal by-products of metabolic processes. They are characterized by heterogeneous properties and have a plethora of downstream effects, depending on their concentrations. Under physiological conditions the continuous production and detoxification of cellular ROS lead to a tightly controlled and well-balanced redox status. On the other hand, an imbalance between ROS production and removal results in the accumulation of ROS in the cells and leads to oxidative stress. In order to maintain the redox balance, cells activate some ROS scavenging systems, mainly composed of antioxidant enzymes and non-enzymatic ROS scavengers. Common enzymes that are involved in the detoxification process of ROS are superoxide dismutase, catalase, peroxiredoxins, and glutathione peroxidases. For their reducing power, glutathione and NAD(P)H are the most well-known electron donors [32].

ROS and associated oxidative stress have been historically considered harmful to the cell as they can damage cellular DNA, oxidize fatty acids and aminoacids. The effects of this oxidation lead to tissue destruction associated with various diseases.

TME is characterized by elevated ROS levels that trigger oxidative stress, deeply affecting tumor progression and metastasis. This condition results from increased basal metabolic activity, mitochondrial dysfunction, peroxisome activity, uncontrolled growth factors and cytokines signalling, and oncogene expression [30]. On the other hand, some ROS source enzymes, as NADPH oxidase (NOXs), cyclooxygenases (COXs), or lipoxygenases (LOXs)s, present higher enzymatic activity in TME [33,34]. Thus, the adaptations to oxidative stress, together with the intrinsic metabolic reprogramming of cancer cells lead to profoundly altered ROS production and sustained oxidative stress in tumor tissue [35,36]. It is important to underline that cells respond to ROS burst activating the detoxification systems with an increase of glutathione and NAD(P)H synthesis that result in a more reducing environment. As a consequence, oxidant-sensitive transcription factors like HIF-1, NF-κB or STAT3 become active and play a mandatory role in eliciting a pro-migratory and pro-inflammatory response in cancer cells [37,38,39,40].

These signaling pathways in TME contribute to render cancer cells even more resistant to a variety of stresses including anticancer drug exposure or mechanical forces when entering the bloodstream [16,41].

## 3. Tumor Dormancy and the Interplay with the Tumor Microenvironment

Metastatic disease rather than primary tumor is one of the major causes of death among cancer patients. In some instances, this occurs shortly after primary tumor detection and treatment because tumor cells are already expanding at the moment of the diagnosis. However, in many types of tumors, metastatic diseases occur years or decades after tumor resection. The appearance of tumor relapse after a prolonged time is explained by the survival of disseminated tumor cells (DTCs) in a dormant state. These cells survive in a quiescent state and remain undetected for long periods, explaining the prolonged asymptomatic residual disease and treatment resistance.

The period between primary tumor detection and metastatic relapse is often defined as tumor dormancy, a poorly understood stage in cancer progression in which cells are characterized by mitotic cycle arrest in G0/G1 phase and low metabolism [42]. This state is reversible, and the dormant cells can re-enter in the cell cycle to proliferate again under certain conditions, such as induction by growth factors, cytokines, nutrients, or chemical agents.

Dormant cells present different genes expression profiles respect to parental cells. As described by Kim et al., the up- and down-regulation of some genes is an important event in the mechanisms of dormancy onset and maintenance of dormancy [43]. The manipulation of the expression of dormancy-associate genes has been proposed as new therapeutic approach. Recently, Tiram et al. validated the role of two genes from signature, thrombospondin-1 (TSP-1) and epidermal growth factor receptor (EGFR), as regulators of glioblastoma dormancy and explored their therapeutic potential in glioblastoma treatment [44].

Tumor dormancy results from tumor cell growth arrest (cellular dormancy) and from mechanisms that antagonize the expansion of a dividing tumor cell population (tumor mass dormancy). The first one can occur when tumor cells enter a state of quiescence. In this regard, the absence of proliferation in dormant cells has been attributed to the up-regulation of cell cycle inhibitors such as p21 and p27.

Conversely to single cell dormancy, tumor mass dormancy is characterized by proliferating cells able to form micrometastatic lesion that does not expand beyond a certain size. It seems to be caused by a balance of cells proliferation and apoptosis, regulated by pro-angiogenic proteins and angiogenic inhibitors produced by tumor and stromal cells, as well as immunological switches. This phenomenon is also termed angiogenic dormancy [45]. According to this definition, Naumov et al. sustain that a failure to activate the angiogenic switch contributes to maintain a group of cells in a dormant state [46]. Indraccolo et al. reported that a short-term perturbation in a transient angiogenic burst could be sufficed to interrupt tumor dormancy [47]. In this regard, therapeutic strategies to target tumor vasculature, such as anti-VEGF drugs, are the current approaches to oppose dormant cells.

Tumor mass dormancy can be also maintained by the immune system that controls and does not completely eliminate malignant tumor growth. This process is termed immunologic dormancy, and given that tumor cells are inherently genetically unstable, the strong immune pressure placed on tumor cells in equilibrium makes them susceptible to acquire mutations that may allow for immune evasion. These adapted tumors frequently defect in antigen presentation, processing, or both, through the loss of major histocompatibility complex (MHC) class I or latent membrane protein (LMP)-family molecules, rendering them undetectable by the adaptive immune system. They are also capable of establishing a global immunosuppressive state in the TME by secreting some cytokines, such as TGF-β and VEGF, or by recruiting immunosuppressive cell type, including T regulatory cells and myeloid-derived suppressor cells (MDSCs). These recruited cells contribute to the anti-inflammatory cytokine production and suppress the anti-tumorigenic capacities of the other immune cell types. Once micrometastases overcome dormancy, they become receptive to signals and cell types within the TME to support their expansion. Thus, controlled immune activation, marked by an induction of T cells, is a promising avenue to force tumor dormancy [45].

To date, little is known regarding tumor dormancy and the biology of dormant cells. Nevertheless, it is clear that one major driving force for tumor dormancy is cellular environment, which poses a challenge to the cells regarding survival and proliferation. Indeed, cells enter or escape dormancy according to restrictive or permissive microenvironment, adapting themselves to better serve its needs. In the restrictive TME, one of the early responses of tumor cells is to reduce growth and the rate of oxygen consumption. Using immunocytochemical techniques, some researchers have demonstrated that hypoxic tumor cells are in a non- or slow-proliferating state and a majority of these cells are negative for proliferation markers, such as Proliferating Cell Nuclear Antigen (PCNA) or Bromodeoxyuridine (BrdU). According to these evidences, the link between cellular dormancy and TME is highlighted by the fact that if the dormant DTCs are originated from the hypoxic niche of the primary tumor, they may be already pre-programmed to be growth-arrested and enter a dormant state before to reach other sites. On the other hand, the inductor of dormancy can be the restrictive microenvironment of the target organs where the DTCs arrive.

However, whether hypoxia signaling prepares a “metastatic soil” in distant organs to remain in a prolonged state of dormancy is not well clarified. However, in several different types of cancers, the bone marrow is a common homing organ for DTCs. In most studies, DTCs are detected in the hypoxic regions of bone marrow and persist there over many years with the potential to recirculate to other organs.

A recent study demonstrated that hypoxic microenvironments in the primary tumor give rise to a subpopulation of DTCs programmed to become dormant [48]. In addition, hypoxia is considered to be a major feature of the TME and is a potential contributor to the CSC phenotype and its enhanced tumorigenicity. This means that hypoxia may contribute to cancer dormancy through different pathways that may be interconnected [49,50]. Moreover, tumor cells adaptation to hypoxic TME is regulated by the expression of more than a hundred genes involved in various biological processes [51,52,53,54,55]. One of them is the epithelial-mesenchymal transition (EMT) where the cancer cells lose the epithelial phenotype and acquire mesenchymal features [56,57,58].

Hypoxia induces EMT by up-regulation or repression of EMT-associated transcription factors such as HIF1-α, NF-Κβ and Notch [59,60]. In particular, the best characterized of them, HIF-1α, induces EMT through the induction of transcription of SNAIL, ZEB1, TWIST and TCF3 [61,62].

EMT induced by hypoxia is a key factor in cancer resistance to apoptosis, to chemotherapy and to the immune response. EMT is one of the initiating events required for the migration into blood vessels of DTCs. Indeed, many studies have demonstrated the hyperexpression of EMT markers in DTCs [63,64].

Hypoxia and HIFs are known to have a role in promoting tumor cell dedifferentiation toward a stem-like phenotype. In order to maintain the self-renewal propriety, hypoxia and HIFs induce the expression of OCT4, SOX2 and NANOG genes [65,66,67].

### Signaling Mechanisms in Tumor Dormancy

To date, several cancer cell-intrinsic pathways that lead to cellular dormancy have been described. The first signaling mechanism that has been connected with cell proliferation and DTC dormancy was the balance between the activities of the mitogen-activated protein kinases (MAPKs) ERK1/2 and p38 [68]. In particular, the switch toward phosphorylation of ERK1/2 favors proliferation, while predominant phosphorylation of p38 lead to quiescence. It is now well accepted that in a permissive microenvironment, the interaction between DTCs and extracellular matrix as well as stromal cells results in the activation of mitogenic signaling (high p-ERK/p-p38) that promote cell growth. Conversely, in restrictive microenvironment, where the activation of stress signaling occurs, low p-ERK/p-p38 ratio represents the molecular switch for induction of a prolonged phase of dormancy [69]. The activities of these kinases were found to be driven by the interaction of urokinase-type plasminogen activator receptor (uPAR), α5β1 integrin, fibronectin focal adhesion kinase (FAK) and Src-kinases. It has been demonstrated that proliferating cells are characterized by high expression of uPAR that lead to activation of Src-kinase resulting in the ERK1/2 phosphorylation. Increased level of fibronectine also results in ERK1/2 phosphorylation. Conversely, p38 phosphorylation is favored when uPAR expression is lost and fibronectin is absent [70,71,72].

Recently, it has been shown that transforming growth factor β2 (TGFβ2) activates p38 in cancer cells disseminated to bone inducing dormancy. This correlates with up-regulation of the proliferation inhibitor p27 and down-regulation of cycling-dependent kinase 4 (CDK4). In this regard, an up-regulation of TGFβ2 and p27 expression induced by hypoxia has been shown in dormant cancer cells, confirming a key role of hypoxia on dormancy [73].

**Figure 4:**
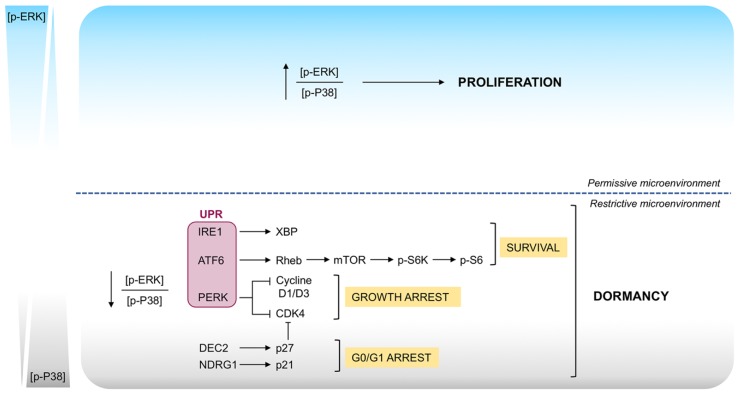
Signaling pathways involved in the switch between proliferation and dormancy. Proliferation to dormancy switch seems to be mediated by interactions between surface receptors such as UPR and integrins, mitogenic signaling from the Ras-extracellular signal-regulated kinase (ERK) pathway, and stress induced signaling from the p38 pathway. In particular, the switch toward phosphorylation of ERK1/2 favors proliferation, while predominant phosphorylation of p38 leads to a G0/G1 arrest, growth arrest and survival that result in dormancy.

Moreover, phosphorylation of p38 leads to the activation of the unfolded protein response (UPR) pathway, which promotes cells survival and dormancy. Some studies from Aguirre-Ghiso laboratory highlight that, although all the three sensors of UPR, PERK, ATF6, IRE1 are activated in dormant human epidermoid carcinoma HEp3 cell, only PERK activation contributes towards the growth arrest of cells [70,74,75,76]. This occurs by attenuating translation of G1-S transition regulators such as cyclin D1, D3 and CDK4. On the other hand, the activation of both ATF6 and IRE1 are required for the basal adaptation and cell survival in restrictive microenvironment. This occurs in part via the ATF6 mediated Rheb induction and a strong inhibition of mTOR signaling (Figure 4) [77].

**Figure 5:**
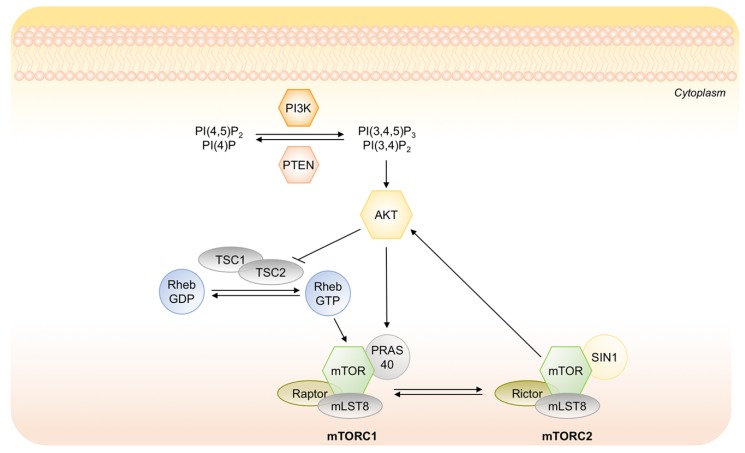
Schematic representation of the PI3K/AKT signaling cascade and its main downstream effectors. Upon stimulation, active PI3K phosphorylates phosphatidylinositol 4 phosphate PI(4)P and phosphatidylinositol (4,5)-bisphosphate (PIP_2_) at their 3 position, generating PI(3,4)P and PI(3,4,5)P_3_. The tumor suppressor PTEN inhibits PI3K activity. Phosphorylated PI(3,4)P_2_ and PI(3,4,5)P_3_ activate phosphorylation of AKT with mTORC2.

Recently, it has been demonstrated the key role of the suppression of PI3K/AKT pathway in the induction of tumor dormancy as well as in the survival disseminated tumor cells [69]. PI3K/AKT signaling regulates cell proliferation, survival and metabolism in cancer cells and it is frequently constitutively activated in multiple human cancers [78]. Under a prolonged lack of nutrients, the suppression of AKT activity is necessary for preserving the energy source, decreasing energy demand and activating a strategy of cancer cells to survive in a chronically deteriorated microenvironment [79]. The mechanism by which AKT activity is suppressed is not completely understood. It may involve different steps in the PI3K/AKT signaling such as inappropriate activation of receptor tyrosine kinase (RTK), inactivation of upstream kinases, i.e., PI3K, activation of phosphates PTEN or/and inactivation of mTORC1 (Figure 5). PI3K/AKT axis directly inhibits glycogen synthase kinase 3-beta, which normally suppresses proliferation, and activates the canonical cell cycle pathway. Conversely, the suppression of AKT that occurs in dormant state, allows to activate both CDKs inhibitors p21 and p27 that arrest cell cycle, and the autophagic machinery that protects cells during starvation or stress condition [80].

## 4. Cancer Stem Cells in Tumor Microenvironment

Recent studies indicate the presence of a small, intra-tumoral subpopulation of tumor-initiating cancer cells with deregulated stem-cell-like properties that enable them to be resistant to conventional therapies. These tumorigenic cancer cells, called cancer stem cells (CSCs), may be the linchpins of disease recurrence and may significantly contribute to metastasis [2].

Specifically, CSCs exhibit a functional stemness signature comprising self-renewal and ability to differentiate into multiple cancer cells [81]. Self-renewal is a characterized mitotic cell division in which a stem cell originates one or two undifferentiated cells perpetuating the stem cell pool. On the other hand, the stem cells differentiate into more specialized cell types generating all the diverse cellular phenotypes of the primary lesion [2]. Evidence supports the vital role of this subset of cells in the initiation and maintenance of a tumor in addition to their capability to dictate invasion, metastasis, heterogeneity, and therapeutic resistance.

### 4.1. Cancer Stem Cells Markers

Since CSCs are endowed with enhanced tumorigenic potential and multidrug resistance, their specific elimination may represent one of the most important challenges of current cancer research. To achieve better understanding and treatment of CSCs, we must identify better the markers of stemness in order to isolate CSCs and analyze their biological characteristics to target them efficiently for therapeutic purposes. Therefore, the identification and isolation of these CSCs using putative surface markers have been a priority of research in cancer. However, the definition of specific CSCs surface markers in all cancer types requires further investigation and effort because the heterogeneity among tumors makes it difficult.

CSC markers must be clearly defined for each tissue, and investigation is needed to determine combinations of target antigens that show high co-expression of surface markers. Some molecules (such as CD133, CD44, ABCG2, ALDH) are already used as biomarkers of some kind of cancer stem cells, but many other cell surface proteins have been identified as the marker of many tumors in the recent years. Identification and checking of the expression of new CSC markers could amplify combinatorial targeting strategies with well-matched target antigens enhancing the therapeutic efficacy. Haubner et al. indicate that combinatorial targeting of CD33/TIM3 or CLL1/TIM3 may enhance therapeutic efficacy without aggravating toxicity in immunotherapy of AML Leukemia [82]. Skoda et al. emphasize the need for further studies that would investigate whether CD24+/CD44+/EpCAM+/CD133+ phenotype specifically identifies pancreatic CSCs [83].

CSCs selection and evolution are influenced by TME that provides a breeding ground to maintain a more stem-like and undifferentiated phenotype. For instance, hypoxia exerts a selective pressure on the CSCs population contributing directly to the development of more aggressive cancer cells and resistance to therapies [84,85]. Recently, it has been demonstrated that prolonged hypoxia exposure results in the induction of genes essential for stem cell function such as Oct4, Nanog and c-Myc. These genes are regulated by HIFs, in particular the isoform HIF-2α, which is specifically involved in the self-renewal and multipotency of CSCs. Molecular database analyses revealed that HIF-2α expression correlates with poor outcome of patients with metastasis and represent a promising target for the eradication of CSCs in cancer therapy [86].

### 4.2. Tumor Dormancy and Cancer Stem Cells

There is a tight and complex relationship between stemness and dormancy even if the ability of CSCs to proliferate and initiate robust tumor outgrowth seems to be incompatible with a cancer dormant state. Elegant studies provide strong evidence for the existence of dormant cells as a subpopulation of CSCs in different tumors such as breast, colon, pancreas and ovary [87,88,89,90,91]. Nevertheless, it is important to note that not all dormant cells have the stem like properties of self-renewal and differentiation [92].

Unlike dormant tumor cells that can remain quiescent for several years, it is not clear if CSCs undergo so long phase of quiescence. However, it is well described that these dormant CSCs exploit phases of cellular dormancy to ensure tumor maintenance and survival in harmful TME, including growth-inhibitory niches and cytotoxic milieus.

It is clear from the previous discussion that hypoxic TME maintains an undifferentiated and quiescent state of CSCs and, since hypoxia promotes tumor dormancy, it could be one of the key links between the dormancy and the stemness. How the hypoxia triggers CSCs populations towards quiescence is still unclear even if some improvements are carried out. It has been demonstrated that slow-cycling CSCs are more likely localized in the low oxygen area of the tumor, away from the blood vessels whereas the fast-cycling cells with limited self-renewal capacities, reside in areas much closer to the vasculature [93].

Moreover, several studies have unraveled intriguing parallels between the mechanisms regulating CSCs behavior and angiogenic control of tumor dormancy. While CSCs might survive in the angiostatic environments associated with dormancy, they also promote cancer vascularization in settings of tumor outgrowth [94,95]. Since CSCs own the ability to promote tumor progression by triggering angiogenic responses, they are likely to represent the dormant tumor populations ultimately responsible for delayed cancer recurrences [93].

Another direct link between CSCs and dormant tumor populations is their converging to survive cancer therapy. Indeed, dormant cells are often spared by current treatment modalities, as determined in both animal models and clinical disease [96,97]. Similarly, CSC frequencies are enhanced in metastatic tumor recurrences post-therapy compared to those in pre-therapy samples. Importantly, therapeutic refractoriness of tumor dormant cells is attributable to several resistance mechanisms also intrinsic to the CSCs compartment, including impairment of cancer apoptotic pathways, alterations of cell cycle checkpoints, and reduced drug accumulation [98].

Taken together these findings highlight the critical importance of TME in the biology of CSCs that creates a strong relationship with tumor dormancy.

However, in light of the intriguing discussion between CSCs biology and the mechanisms controlling tumor dormancy, insights from CSCs biology could help the future research on tumor dormancy.

## 5. Autophagy in Tumor Microenvironment

Autophagy, or type II programmed cell death, is a catabolic process, evolutionarily conserved and genetically controlled, whereby cells self-digest intracellular organelles to remove those with compromised function and to maintain cell homeostasis. However, autophagy can also be considered a temporary survival mechanism during periods of starvation where self-digestion offers an alternative energy source and may facilitate the disposal of unfolded proteins under stress conditions. The catabolic function provided by autophagy is thereby suppressed once the external nutrient supply is adequate to support cellular metabolism. Besides starvation, autophagy can be also activated by other physiological stress stimuli, such as hypoxia, endoplasmatic reticulum stress, high temperature, hormonal stimulation or pharmacological agents [99].

Depending on the mode of cargo delivery to the lysosome, autophagy can be subdivided into three subtypes: chaperon-mediated autophagy, microautophagy and the most studied process named macroautophagy [100]. Chaperone-mediated autophagy involves the direct translocation of cytosolic proteins across the lysosomal membrane, by molecular chaperones (e.g., the 70 kDa heat shock cognate protein, Hsp70). Microautophagy involves inward invagination of lysosomal membrane, which delivers a small portion of cytoplasm into the lysosomal lumen. Macroautophagy is the most efficient autophagic clearance mechanism and the most common type of autophagy, therefore, hereafter macroautophagy will be referred to as autophagy. Macroautophagy is a process conserved by yeast to mammals and is mediated by a double-membrane special organelle termed autophagosome. Upon induction, a small vesicle (phagophore) elongates and encloses a portion of cytoplasm, which results in the formation of the autophagosome. Then, the outer membrane of the autophagosome fuses with a lysosome to form an autolysosome, leading to the degradation of the enclosed materials together with the inner autophagosomal membrane. Aminoacids and other small molecules that are generated by autophagic degradation are delivered back to the cytoplasm for recycling or energy production [101]. Specifically, the process of autophagosome formation involves two major steps, nucleation and elongation of the isolation membrane, in which different proteins, such as the products of AuTophaGy-related (Atg) genes and microtubule-associated protein light chain 3 (LC3) are involved [102]. LC3 is one of the best characterized protein on autophagosome, and therefore, it serves as widely used marker of autophagy [103]. It is expressed in cells as a full-length cytosolic protein that, upon autophagy induction is proteolytically cleaved, to generate LC3-I that is conjugated with phosphatidylethanolamine and forms LC3-II (Figure 5) [104,105,106].

**Figure 6:**
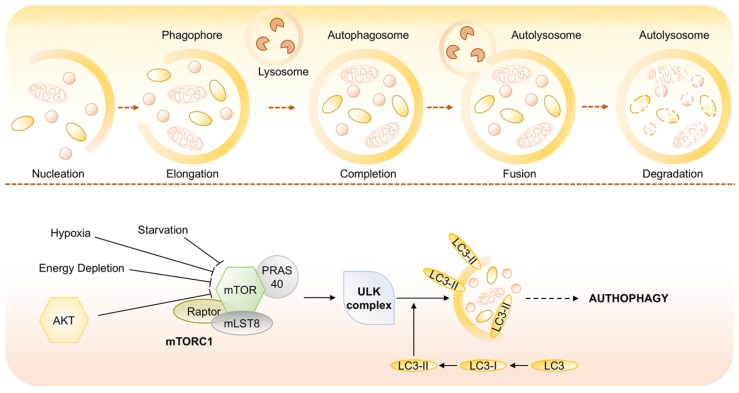
Molecular signaling pathways regulating autophagy. Autophagy is mediated by a double-membrane special organelle termed autophagosome that, upon induction, fuses with a lysosome to form an autolysosome, leading to the degradation of the enclosed materials. The central signaling molecule in determining the levels of autophagy in cells is the mTOR kinase that is recognized as sensor that coordinately regulates the balance between growth and autophagy in response to cellular physiological conditions and environmental stress and likely mediates its effects on autophagy through inhibition of ATG1/ULK-1/-2 complexes at the earliest stages in phagophore formation from lipid bilayers.

Among the numerous components involved in the regulation of autophagy, the mammalian target of rapamycin (mTOR) is now recognized as sensor that co-ordinately regulates the balance between growth and autophagy in response to cellular physiological conditions and environmental stress [107]. mTOR is a serine/threonine protein kinase that belongs to the phosphatidylinositol kinase-related kinase (PIKK) family. The inhibition of mTOR activity through Akt and MAPK signaling, under nutrient starvation, induces autophagy as well as negative regulation of mTOR, through AMPK and p53 signaling, promotes it (Figure 6) [108].

Under physiological conditions, autophagy has a number of other vital roles such as the maintenance of the aminoacid pool during starvation, prevention of neurodegeneration, antiaging function, clearance of intracellular microbes, and regulation of innate and adaptive immunity [100,109].

In addition to the physiological roles of autophagy, many reports describe its controversial involvement in the genesis and the progression of cancer [110,111,112]. Indeed, autophagy acts either as a tumor suppressor, by preventing the accumulation of damaged proteins and organelles, or as a mechanism of cell survival that promote and maintain the growth of established tumors [113].

Activated AKT inhibits TSC2 via phosphorylation and inactive TSC1/2 is unable to bind RAS homolog enriched in brain (RHEB), which subsequently enables the activation of mTORC1 at the surface of lysosome.

### Autophagy Sustains Survival of Dormant Cancer Cells

A potential role of autophagy in dormancy was originally evidenced in *C. elegans* during a dormancy-like state, where larvae are exposed to hostile microenvironment. In this model, *C. elegans* activated autophagy that could promote survival during quiescent states [114].

Since autophagy is activated in response to changes of the microenvironment, it could be interesting to investigate the mechanisms that induce autophagy and allow to the survival and the maintenance of the dormant state in tumor cells. Although the efforts to show through in vitro or in vivo models, the role of autophagy in dormancy and the mechanisms that are activated during this state remain largely discussed. However, studies in models of breast cancer cells suggest that the decreased mitogenic signaling of β1-integrin in dormancy may stimulate autophagy [115]. Moreover, it has been shown that dormant cancer cells activate p27 that is involved in cell cycle arrest and directly induces autophagy to facilitate cells survival in response to growth factor withdrawal [116,117].

It would be critical to determine whether autophagy plays a dormant or survival-inducing role, or both, in quiescence tumor cells. If autophagy induces a pro-survival state, then strategies to block it could eradicate dormant cells. In case that it contributes to both quiescence and survival, then more detailed analysis of these pathways would be required to reveal ways to inhibit only the survival signals without interrupting quiescence [118].

## 6. Models for the Study of Dormancy

FDA approved cancer drugs are usually optimized to be highly effective in vitro using cancer cells monolayers and in vivo using mouse xenograft cancer models [119,120,121]. However, considering the complexity of tumors, there is a gap between these two models. In fact, the design of innovative therapies for effective cancer therapy require adequate preclinical models that mimic TME. With that purpose, cell and tissue engineered tumor models have been gaining attention since they can recapitulate more closely the TME to which the cells within the tumor are exposed (e.g., survival, proliferation, gene expression heterogeneity and multidrug resistance), also enabling the control of environmental factors and measurement of cell responses [122,123]. Experiments conventionally start on 2D models, providing initial improvements using monocultures of commercial/immortalized cell lines, in a simple, convenient and relatively reproducible way. These 2D cultures can be improved by using co-cultures of different cell types to better resemble human tissues cell-to-cell communications [124,125]. To improve even more tissue complexity (mechanical and biochemical signals), mimicking the tumor architecture, 3D (co-)culture systems have been employed [119,126]. The development of more biologically relevant in vitro tumor models using 3D approaches not only results in improved translation but also contributes to reducing animal testing (three Rs politics) required by the pharmaceutical industry and governmental institutions [126,127].

However, in vitro models to induce dormancy in tumor cells are still few. Those models have been classified according to the dormancy-inducing sources that are used [128]. Herein, we will focus on the in vitro dormancy models that use hypoxia to mimic the stressful TME. The simplest model to achieve hypoxic 2D cultures is to incubate cells in a hypoxic chamber. Louie et al. have shown that, exposing the metastatic human breast cancer cell lines MDA-MD231 and BCM2 to hypoxia (1% O_2_)/reoxygenation cycles, a unique sub-population can be selected. This population is able to quickly form colonies and present both stem-like and EMT (epithelial mesenchymal transition) phenotypes. Moreover, CSCs are highly tumorigenic when injected into immune-deficient mice [129]. Recently, we have obtained and characterize the chronic hypoxia resistant MDA-MB-231 (chMDA-MB-231) cell line. ChMDA-MB-231 cells are the sub-population selected from MDA-MB-231 exposed to at least three hypoxia/reoxygenation cycles that present stem-like phenotype and spheroid forming ability. We have shown how this hypoxia-resistant breast cancer cell line manage to survive to hypoxic/reoxygenation stress by entering into a reversible dormant state defined by low metabolism, arrest in G0/G1 phase and decreased proliferation rate. Furthermore, we proposed a survival mechanism founding that in chMDA-MB-231 cells autophagy is strongly induced [84]. It has been shown that also a pancreatic cell line, AsPC-1, is uncommonly able to survive under chronic hypoxia (1% O_2_) for weeks entering in a state of dormancy. It has been shown that the induction of dormancy using hypoxia can be observed also in primary cultured cells derived from lung, colorectal and urothelial cancer, but this model requires both chronic hypoxia and growth factor deprived conditions [79]. Besides using the hypoxic chamber, it is possible to obtain a hypoxia-mimicking microenvironment adding iron-binding/substitute agents, such as di cobalt chloride (CoCl_2_), in cell culture medium. This hypoxia-mimetic agent is able to stabilize cytosolic expression of HIF1α, one of the major regulators of hypoxia signaling, inhibiting its degradation. In vitro cancer cells dormancy hypoxia-regulated can be induced and maintained by addition of CoCl_2_ in cultures of breast cancer MCF-7, MDA-MB-231 and ovarian cancer OVCAR-3 cell lines. The dormancy responses obtained in this model is comparable to cells maintained in 0.1% O_2_ conditions [130]. Finally, a different in vitro model of hypoxia-induced tumor dormancy can be achieved also imposing a diffusion-limited hypoxia culturing cells in 3D hydrogel systems that, limiting the access of oxygen and nutrients, led to reduced cells proliferation and cellular quiescence. Prostate cancer cells C4-2B, colon cancer cells HCT116, and breast cancer cells MDA-MB-231 have been cultured in a transglutaminase-crosslinked gelatin gel, called Col-gel, for a few days. Increasing culture time, up to nine days, it has been observed a gradual lowering of nutrient diffusion and of oxygen levels at the center of the Col-gel culture causing a higher proliferation of the peripheral cells, that forms spheroids and clusters, and inducing quiescence or necrosis of the inner ones. This system has been also used to mimic the tumor niche in a co-culture model of human squamous carcinoma cells (SCC-71) and bone marrow derived mesenchymal stem cells [131]. As evidenced by these newly established in vitro models, the development of systems to mimic hypoxia-induced tumor dormancy is a functional approach to study the mechanism that regulate both primary and disseminated tumor development.

## 7. Prospective

The hypoxic tumor microenvironment has been recognized as a cause of malignancy or resistance to various cancer therapies. However, the effect of a chronic exposure of the cells to hypoxia and the following reoxygenation cycles remain elusive. Although most of the tumor cells die in chronic hypoxia, some of them can survive for more than several days in a quiescent state. This dormant state is reversible, with tumor cells recovering the ability to self-renew once closed vessels reopen or new vasculatures reach the hypoxic area. Because dormant tumor cells may be the founders of metastasis, one hypothesis is that these cells share stem cell-like characteristics that may be responsible for their long life.

An understanding of the regulatory machinery of tumor dormancy is essential for identifying early cancer biomarkers and could provide a rationale for the development of novel agents to target dormant tumor cells population. The lack of established in vitro models of tumor dormancy represents the main factor that hampers the understanding of dormant cell responses. Indeed, the majority of in vitro hypoxia studies have been carried out exposing cell lines to acute hypoxia (3–24 h), whereas only few reports of chronic hypoxia have been published.
